# New species of the subgenus *Epiphragma* Osten Sacken from China (Diptera, Limoniidae)

**DOI:** 10.3897/zookeys.876.33163

**Published:** 2019-09-18

**Authors:** Bing Zhang, Meng Mao, Ding Yang

**Affiliations:** 1 Department of Entomology, College of Plant Protection, China Agricultural University, Beijing 100193, China China Agricultural University Beijing China

**Keywords:** Limnophilinae, morphology, description, key

## Abstract

Previously 12 species of the subgenus Epiphragma (Epiphragma) Osten Sacken, 1860 were known to occur in China. The following three species are described and illustrated as new to science: E. (E.) acuminatum**sp. nov.**, E. (E.) henanensis**sp. nov.**, and E. (E.) longitubum**sp. nov.**. Epiphragma (E.) insigne van der Wulp, 1878 is reported from China for the first time and is illustrated based on additional morphological characters. An updated key to the species of the subgenus E. (Epiphragma) from China is presented.

## Introduction

The subgenus Epiphragma (Epiphragma) Osten Sacken is the largest subgenus in the genus *Epiphragma*, with 115 known species, and is considered to be monophyletic (Ribeiro 2008). It is distributed worldwide with ten species from the East Palaearctic, 27 species from the Oriental Region, five species from the Nearctic Region, 64 species from the Neotropical Region, and 14 species from Australasian/Oceanian Region (Oosterbroek 2019). It is delimited by the following combination of characters: flagellum with two or more basal segments fused; wing broad, variegated with brown bands, spots; a single supernumerary cross-vein in cell C; posterior margin of tergite 9 with subtrigonal lobe on each side of median notch (Dienske 1987).

The following 12 species were previously known from China: Sichuan: E. (E.) bicinctiferum Alexander, 1935, E. (E.) subobsoletum Alexander, 1936, E. (E.) sultanum Alexander, 1938; Zhejiang, Hubei: E. (E.) evanescens Alexander, 1940; Taiwan: E. (E.) divisum Alexander, 1923, E. (E.) kempi Brunetti, 1913, E. (E.) nymphicum Alexander, 1928; Yunnan: E. (E.) ancistrum Mao & Yang, 2009, E. (E.) breve Mao & Yang, 2009, E. (E.) elongatum Mao & Yang, 2009, E. (E.) mediale Mao & Yang, 2009, E. (E.) yunnanense Mao & Yang, 2009. Of these 12 species, ten are endemic to China, E. (E.) evanescens being also known from Japan and E. (E.) kempi from Japan, India, and Sri Lanka. Here three new species are added to the fauna of China, as well as E. (E.) insigne, known previously only from Indonesia and Malaysia. An updated key to the species of the subgenus E. (Epiphragma) from China is presented.

## Materials and methods

The specimens were studied and illustrated with a ZEISS Stemi 2000-c stereo microscope. Genitalic preparations were made by macerating the apical portion of the abdomen in cold 10% NaOH for 12–15 h. After examination in glycerin, genitalia were transferred to fresh glycerin and stored in a microvial pinned below the specimen. The type specimens of the new species are deposited in the Entomological Museum of China Agricultural University (CAU), Beijing, China.

Terminology of morphological features generally follows that of McAlpine (1981) except wing veins which follow Dienske (1987).

## Taxonomy

### Key to species of subgenus Epiphragma from China

**Table:** 

1	Femora unpatterned (Fig. 7)	**2**
–	Femora patterned (Figs 1–6)	**3**
2	Origin of Rs with short spur (Figs 12, 15); m-cu at basal 1/4 of cell *dm*	**E. (E.) divisum (Alexander, 1923)**
–	Origin of Rs without spur (Fig. 14); m-cu at basal 3/4 of cell *dm* (Fig. 14)	**E. (E.) insigne (van der Wulp, 1878)**
3	Femora black or yellow with one subterminal ring	**4**
–	Femora yellow with two dark brown rings (Figs 1–6)	**8**
4	Femora black with narrow yellow subterminal ring; verticils on antenna shorter than segments	**E. (E.) sultanum (Alexander, 1938)**
–	Femora yellow with darker subterminal ring; verticils on antenna longer than segments	**5**
5	Femora with broad black subterminal ring; prescutum with narrow black median stripe obsolete for some distance before suture	**E. (E.) nymphicum (Alexander, 1928)**
–	Femora with pale brown subobsolete ring before tip; prescutum not as above	**6**
6	Dark pattern of wing without narrow dark brown margin	**E. (E.) subobsoletum (Alexander, 1936)**
–	Dark pattern of wing with narrow dark brown margin	**7**
7	R_2+3+4_ nearly straight, in virtual longitudinal alignment with Rs; *m-cu* strongly sinuous	**E. (E.) evanescens (Alexander, 1940)**
–	R_2+3+4_ not as above; *m-cu* straight	**E. (E.) kempi (Brunetti, 1913)**
8	Dark pattern of wing without narrow dark brown margin (Fig. 13)	**9**
–	Dark pattern of wing with narrow dark brown margin (Figs 12, 14, 15)	**10**
9	Spur at origin of Rs almost obsolete; apical half of interbase like a slender rod bent at a 90-degree angle from thickened base	**E. (E.) mediale (Mao & Yang, 2009)**
–	Spur at origin of Rs distinctly longer (Fig. 13); apical half of interbase finger-shaped (Figs 26, 27)	**E. (E.) henanensis sp. nov.**
10	Interbase very long, at least as long as gonocoxite	**11**
–	Interbase relatively short, shorter than gonocoxite	**15**
11	Second ring of femora at tip (Figs 1, 2, 5, 6)	**12**
–	Second ring of femora near tip (Figs 3, 4)	**14**
12	Aedeagus 4/5 as long as gonocoxite (Figs 38, 39)	**E. (E.) longitubum sp. nov.**
–	Aedeagus less than 4/5 as long as gonocoxite (Figs 20, 21)	**13**
13	Crossvein *m-cu* at basal 1/4 of cell dm; interbase finger-shaped	**E. (E.) elongatum (Mao & Yang, 2009)**
–	Crossvein *m-cu* at basal 1/3 of cell dm (Fig. 12); interbase S-shaped (Figs 20, 21)	**E. (E.) acuminatum sp. nov.**
14	Base of interbase subtriangular; inner gonostylus with several setae at tip	**E. (E.) bicinctiferum (Alexander, 1935)**
–	Base of interbase triangular, connected with one small ellipsoidal hole; inner gonostylus without setae at tip	**E. (E.) yunnanense (Mao & Yang, 2009)**
15	Pronotum light brown without any black stripe; hyaline spots of wing rather small; median area of tergite 9 with small prominence; inner gonostylus setae, with tip curved into small spine	**E. (E.) ancistrum (Mao & Yang, 2009)**
–	Pronotum brown with one black stripe; hyaline spots of wing rather large; median area of tergite 9 plane without small prominence; inner gonostylus with obtuse tip and several setae at base	**E. (E.) breve (Mao & Yang, 2009)**

### Epiphragma (Epiphragma) acuminatum sp. nov.

Taxon classificationAnimaliaDipteraLimoniidae

7865B404-5BA2-5F2A-876F-AE5DAAD9C516

http://zoobank.org/8D097E94-414F-4B15-8E28-99EF59F9637D

Figs 1 , 2 , 8 , 12 , 16–21

#### Diagnosis.

Generally brownish yellow with pale gray pruinosity. Femora yellow with two dark brown rings, first at basal 2/3, second at tip, broader. Wing brownish hyaline with conspicuous brown pattern, chiefly spotted, with narrow dark brown margin, brownish hyaline areas large. Origin of Rs obtuse and curved with short spur; *m-cu* at basal 1/3 of cell *dm.* Interbase with long and slender rod beyond base, longer than gonocoxite, apex with sharp point; whole slender rod S-shaped. Aedeagus 1/2 as long as gonocoxite, sword-tip shaped.

#### Description.

**Figures 1–6.**
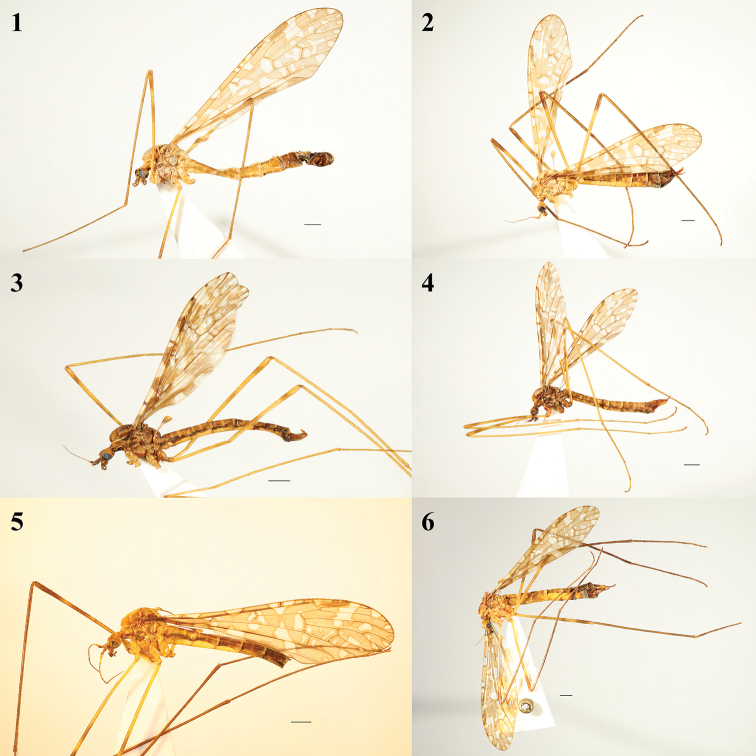
**1, 2**Epiphragma (Epiphragma) acuminatum sp. nov. **3, 4**Epiphragma (Epiphragma) henanensis sp. nov. **5, 6**Epiphragma (Epiphragma) longitubum sp. nov. **1, 3, 5** male habitus, lateral view **2, 4, 6** female habitus, lateral view. Scale bars: 1 mm.

**Male.** (n = 3): Body length 11.5–13.0 mm, wing length 12.0–14.0 mm, antenna length 2.5–2.7 mm. Head (Figs 1, 8). Brownish yellow with pale gray pruinosity except orbit pale yellow. One median tubercle between eyes. Setae on head black. Scape and pedicel brown, flagellum 13-segmented with two basal segments fused, fusion-segment yellow, succeeding segments brownish, flagellomeres cylindrical, apical segments elongate, with longer verticils. Proboscis brownish black with black setae; palpus black with black setae.

Thorax (Figs 1, 8). Generally brownish yellow with pale gray pruinosity. Pronotum and prescutum brownish yellow. Prescutum with four black stripes, intermediate pair long and almost extended to transverse suture. Scutum brownish black, intermediate brownish yellow, scutellum and mediotergite brownish yellow. Pleura brown, variegated by brownish yellow areas. Setae on thorax brown. Coxae and trochanters yellow; femora yellow with two dark brown rings, first at basal 2/3, second at tip, broader; tibiae brown with one spur; tarsi brown. Setae on legs brown except coxae with brownish yellow setae. Wing (Figs 1, 12). Brownish hyaline with conspicuous brown pattern, chiefly spotted, with narrow dark brown margin, brownish hyaline areas large. Base of wing brown, connected with two large ocellate circles with flattened tips; one circle along cord connected with second circle. Stigma solidly dark brown. Origin of Rs obtuse and curved with short spur; R_2+3+4_ longer than R_2+3_; *m-cu* at basal 1/3 of cell *dm.* Halter 1.7–1.8 mm long, brownish black except base of stem yellow and apex of knob pale yellow. Setae on wings brownish yellow.

Abdomen (Fig. 1). Tergites 1–5 brownish yellow; tergites 6–9 brownish black. Sternites 1–5 grayish yellow, both sides brownish yellow; sternites 6–9 brownish black. Setae on abdomen brown. Hypopygium (Figs 16–21). Posterior margin of tergite 9 (Figs 16, 19, 20) with subtrigonal lobe on each side of median V-shaped notch. Base of outer gonostylus (Figs 17, 19–21) broad, tip curved into spine. Base of inner gonostylus (Figs 18–21) with setae, tip obtuse and curved up. Interbase (Figs 20, 21) with long and slender rod beyond base, longer than gonocoxite, apex with sharp point; whole slender rod S-shaped. Aedeagus (Figs 20, 21) half as long as gonocoxite, sword-tip shaped.

**Figure 7.**
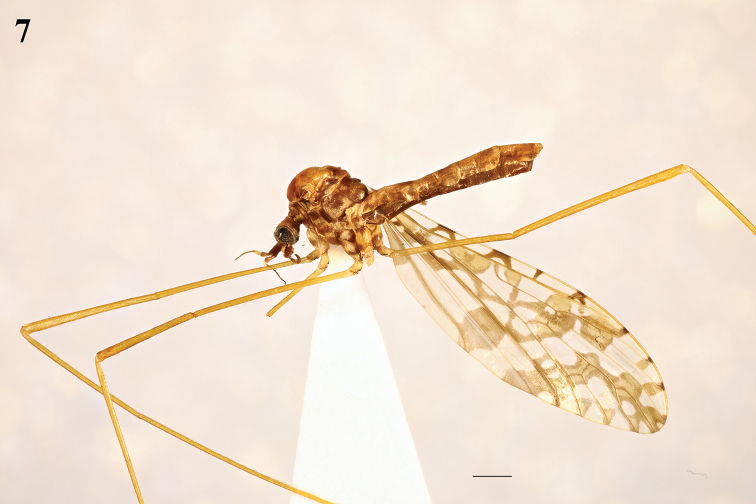
Epiphragma (Epiphragma) insigne (van der Wulp, 1878). Male habitus, lateral view. Scale bar: 1 mm.

**Female.** (n = 19): Body length 12.6–14.0 mm, wing length 12.8–14.2 mm, antenna length 2.5–2.7 mm. Similar to male. Cerci reddish brown; hypovalves brown (Fig. 2).

#### Type Material.

**Holotype** male (CAU), China: Ningxia, Jingyuan, Liupanshan, Liangdianxia, 2007.VI.28, Gang Yao (light trap). **Paratypes**: 2 males, 8 females (CAU), China: Ningxia, Jingyuan, Liupanshan, Liangdianxia, 2007.VI.28, Gang Yao (light trap); 2 females (CAU), China: Ningxia, Jingyuan, Liupanshan, Liangdianxia, 2007.VI.27, Gang Yao (light trap); 8 females (CAU), China: Ningxia Yinchuan Helanshan Suyukou, 2007. VII. 5, Gang Yao; 1 female (CAU), China: Ningxia, Jingyuan, Liupanshan, Liangdianxia, 2007.VII.15, Gang Yao (light trap).

#### Distribution.

China (Ningxia).

#### Etymology.

The specific name refers to the interbase narrowing to a slender point.

#### Remarks.

This new species is somewhat similar to E. (E.) sultanum Alexander, 1938 from China (Sichuan) in having a similar shape of the hypopygium, but it can be separated from the latter in having the femora yellow with two dark brown rings, the origin of Rs obtuse and curved with a short spur, and *m-cu* at basal 1/3 of cell *dm.* In E. (E.) sultanum, the femora are black brown with a narrow yellow ring; the origin of Rs is obtuse and without a spur; and *m-cu* is at basal 1/2 of cell *dm* (Alexander 1938).

### Epiphragma (Epiphragma) henanensis sp. nov

Taxon classificationAnimaliaDipteraLimoniidae

1BFC9217-AB2A-56F1-A949-69A362A9FD88

http://zoobank.org/18B3FC53-9530-4289-9C5E-0E276E1D26F4

Figs 3 , 4 , 9 , 13 , 22–27

#### Diagnosis.

Generally brown with gray pruinosity. Vertex with one median brown line. Femora yellow with two brown rings, first at basal 2/3, second at before tip, longer than yellow tip. Wing brownish hyaline with conspicuous brown pattern, chiefly spotted, without narrow dark brown margin. Origin of Rs obtuse and sharp with long spur; *m-cu* at basal 1/2 of cell *dm.* Interbase with long and slender rod beyond base, almost as long as gonocoxite, apex with sharp point, whole slender rod finger-shaped. Aedeagus 1/2 as long as gonocoxite, sword-tip shaped.

#### Description.

**Male.** (n = 27): Body length 8.5–10.5 mm, wing length 9.6–12.0 mm, antenna length 1.2–1.8 mm. Head (Figs 3, 9). Brownish yellow. Vertex with one brown median line. One median tubercle between eyes. Setae on head black. Scape and pedicel dark brown, flagellum 13-segmented with two basal segments fused, fusion-segment yellow, succeeding segments dark brown, flagellomeres cylindrical, apical segments elongate, with longer verticils. Proboscis brownish yellow with black setae; palpus black with black setae.

Thorax (Figs 3, 9). Generally dark brown with pale gray pruinosity. Pronotum brown with one brownish yellow stripe. Prescutum brownish with four dark brown stripes, intermediate pair long, with lateral, humeral and anterior parts brown. Scutum and scutellum brown. Mediotergite black brown. Pleura brown, variegated by dark brown areas. Setae on thorax black brown. Coxae pale brown; trochanters brownish yellow; femora yellow with two brown rings, first at basal 2/3, second near tip, longer than yellow tip; tibiae brownish yellow with one spur; tarsi brownish yellow with bright yellow tip. Setae on legs black brown. Wing (Figs 3, 13). Brownish hyaline with conspicuous brown pattern, chiefly spotted, without narrow dark brown margin. Base of wing brown, connected with two large ocellate circles with flattened tips; one circle along cord connected with second circle, each tip of vein with brown spot. Stigma solidly dark brown. Origin of Rs obtuse and sharp with long spur; R_2+3+4_ longer than R_2+3_; *m-cu* at basal 1/2 of cell *dm.* Halter 1.1–1.6 mm long, brownish black except base of stem yellow and apex of knob brownish yellow. Setae on wings brownish yellow.

**Figures 8–11.**
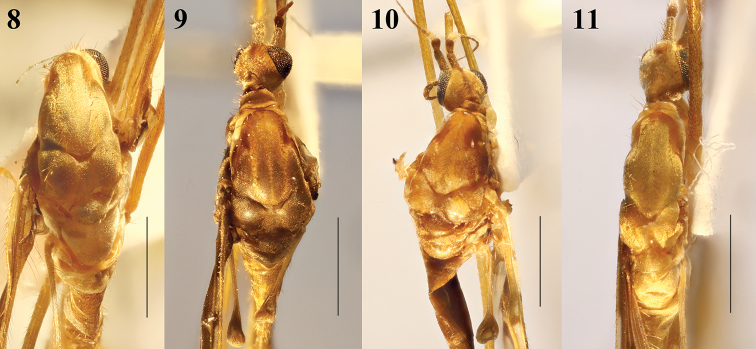
Head and thorax (male). dorsal view. **8**Epiphragma (Epiphragma) acuminatum sp. nov. **9**Epiphragma (Epiphragma) henanensis sp. nov. **10**Epiphragma (Epiphragma) insigne (van der Wulp, 1878) **11**Epiphragma (Epiphragma) longitubum sp. nov. Scale bars: 1 mm.

Abdomen (Fig. 3). Tergites brown with median brownish stripe, both sides with black brown lines. Sternites brownish yellow. Last three segments black brown. Setae on abdomen black. Hypopygium (Figs 22–27). Posterior margin of tergite 9 (Figs 22, 25, 26) with subtrigonal lobe on each side of median V-shaped notch. Tip of outer gonostylus (Figs 23, 25–27) abruptly slender, curved into spine. Inner gonostylus (Figs 24–27) apically obtuse with setae. Interbase (Figs 26, 27) with long and slender rod beyond base, almost as long as gonocoxite, apex with sharp point, whole slender rod finger-shaped. Aedeagus (Figs 26, 27) half as long as gonocoxite, sword-tip shaped.

**Female.** (n = 57): Body length 8.6–12.0 mm, wing length 9.5–12.3 mm, antenna length 1.2–1.8 mm. Similar to male. Cerci reddish brown; hypovalves reddish yellow (Fig. 4).

#### Type Material.

**Holotype** male (CAU), China: Henan, Nanyang, Neixiang, Baotianman, 2004.VII.22, Kuiyan Zhang. **Paratypes**: 1 female (CAU), China: Shaanxi, Foping, Xigou, 2006.VII.27, Yajun Zhu; 2 females (CAU), China: Henan, Nanyang, Neixiang, Baotianman, 2004.VII.22, Hui Dong; 1 female (CAU), China: Henan, Nanyang, Neixiang, Baotianman, 2004.VII.23, Hui Dong; 1 female (CAU), China: Henan, Nanyang, Neixiang, Baotianman, 2008.VIII.10, Ding Yang; 1 female (CAU), China: Henan, Nanyang, Neixiang, Baotianman, 2008.VIII.11, Xingyue; 1 female (CAU), China: Hubei, Shennongjia, Liujiawuchang, 2007.VII.30, Qifei Liu (light trap); 1 female (CAU), China: Hubei, Shennongjia, Liujiawuchang, 2007.VII.31, Qifei Liu (light trap); 1 female (CAU), China: Hubei, Shennongjia, Ping Qian, 2007.VII.25, Qifei Liu (light trap); 1 female (CAU), China: Hubei, Shennongjia, Dalongtan, 2009.VI.27, Qifei Liu (light trap); 2 males, 1 female (CAU), China: Hubei, Shennongjia, Guanmenshan, 2009.VII.2, Qifei Liu (light trap); 20 males, 29 females (CAU), China: Hubei, Shennongjia, Qiangjiaping, 2009.VII.4, Qifei Liu (light trap); 1 male, 2 females (CAU), China: Hubei, Shennongjia, Qiangjiaping, 2009.VII.4, Liang Liang (light trap); 2 males (CAU), China: Hubei, Shennongjia, Caiqi, 2009.VII.14, Qifei Liu (light trap); 1 female (CAU), China: Hubei, Shennongjia, Caiqi, 2009.VII.14, Liang Liang (light trap); 1 male, 13 females (CAU), China: Hubei, Shennongjia, Yinyuhe, 2009.VII.18, Qifei Liu (light trap); 1 female (CAU), China: Hubei, Shennongjia, Yinyuhe, 2009.VII.18, Liang Liang (light trap).

#### Distribution.

China (Henan, Shaanxi, Hubei).

#### Etymology.

The species is named after the type locality Henan.

#### Remarks.

This new species is somewhat similar to E. (E.) elongatum Mao & Yang, 2009 from China (Yunnan) in having a similar shape of the hypopygium, but it can be separated from the latter by the wing pattern without narrow dark brown margin, the origin of Rs with a long spur, and *m-cu* at basal 1/2 of cell *dm.* In E. (E.) elongatum, the pattern of the wing has a narrow dark brown margin; the origin of Rs has a short spur; and *m-cu* is at basal 1/4 of cell *dm* (Mao and Yang 2009).

### Epiphragma (Epiphragma) insigne

Taxon classificationAnimaliaDipteraLimoniidae

(van der Wulp, 1878)

7B45AB4D-DF13-54A7-B902-4F942A7F071F

Figs 7 , 10 , 14 , 28–33

Epiphragma (Epiphragma) insigne van der Wulp, 1878. Tijdschr. Ent. 21: 196. Type locality: Indonesia (Sumatra).

#### Diagnosis.

Generally brown with gray pruinosity. Vertex with one median brown line. Femora yellow without ring. Wing brownish hyaline, with conspicuous brown pattern, chiefly spotted, without narrow dark brown margin; one long irregular hyaline band cross whole wing from tips of veins R_3_, R_4_, ending at cell A_1_; hyaline areas large. Origin of Rs obtuse and curved without spur; R_2+3+4_ three times longer than R_2+3_; *m-cu* at basal approximately 3/4 of cell *dm.* Interbase with base expanded, shorter than gonocoxite, but longer than aedeagus, apical rod slender, C-shaped, bent to sternite, tip curved into slender spine. Aedeagus very short and small.

#### Description.

**Male.** (n = 1): Body length 9.0 mm, wing length 9.8 mm, antenna length 1.7 mm. Head (Figs 7, 10). Brownish yellow. Vertex with one black median line. One median tubercle between eyes. Setae on head black. Scape and pedicel black brown, flagellum 13-segmented with two basal segments fused, fusion-segment yellow, succeeding segments black brown, flagellomeres cylindrical, apical segments elongate, with longer verticils. Proboscis black brown with black setae; palpus blackish brown with black setae.

Thorax (Figs 7, 10). Generally brown with pale gray pruinosity. Pronotum brownish yellow with one dark blackish brown stripe. Prescutum brown with two brownish yellow stripes, one dark brown line at middle. Scutum yellow. Scutellum and mediotergite brownish yellow. Pleura brown, variegated by black brown areas. Setae on thorax brown. Coxae brownish yellow with one dark brown middle ring; trochanters yellow, brown at tip; femora yellow without ring; tibiae yellow with one spur; tarsi yellow. Setae on wings brownish yellow. Wing (Figs 7, 14). Brownish hyaline, with conspicuous brown pattern, chiefly spotted, without narrow dark brown margin; base of wing brown; each tip of vein with brown spot; one long irregular hyaline band across whole wing from tips of veins R_3_, R_4_, ending at cell A_1_; hyaline areas large. Origin of Rs obtuse and curved without spur; R_2+3+4_ three times longer than R_2+3_; *m-cu* at basal approximately 3/4 of cell *dm.* Setae on wings brownish yellow. Halter 1.2 mm long, brownish yellow except base of stem yellow and apex of knob pale yellow. Setae on legs brownish yellow.

Abdomen (Fig. 7). Tergites brownish yellow except tergite 9 yellow. Sternites brown except sternite 9 yellow. Setae on abdomen brown. Hypopygium (Figs 28–33). Posterior margin of tergite 9 (Figs 28, 31, 32) with relatively large subtrigonal lobe on each side of median V-shaped notch. Base of outer gonostylus (Figs 29, 31–33) with setae, tip curved into spine. Inner gonostylus (Figs 30–33) relatively longer with tip obtuse. Interbase (Figs 32, 33) with base expanded, shorter than gonocoxite, but longer than aedeagus, apical rod slender, C-shaped, bent to sternite, tip curved into slender spine. Aedeagus (Figs 32, 33) very short and small.

**Figures 12–15.**
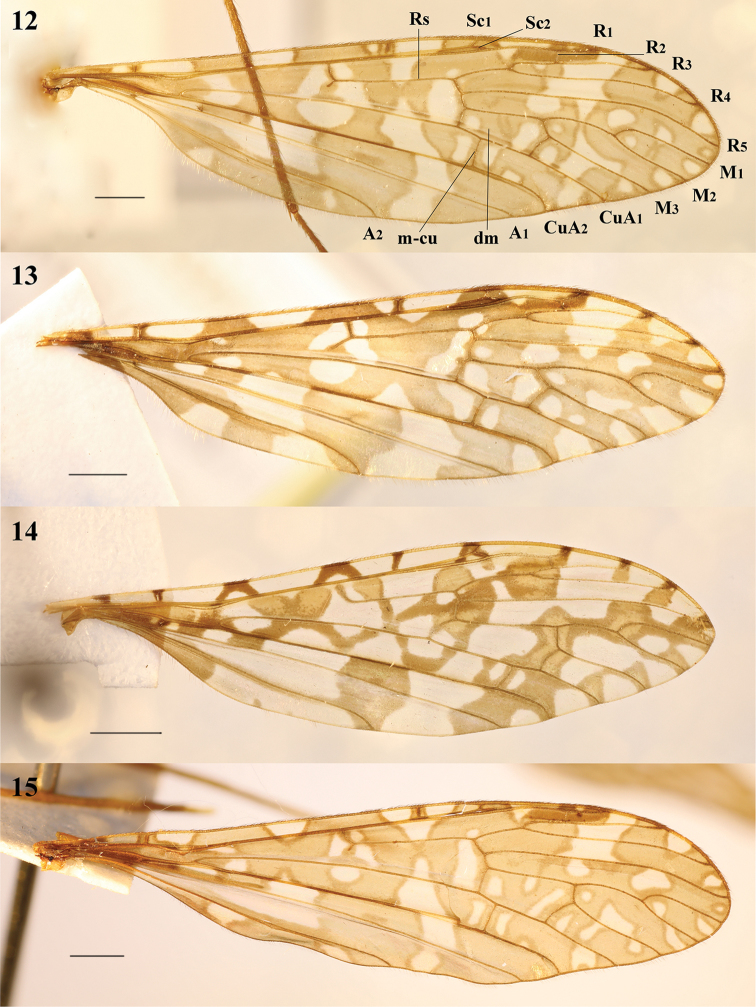
Wing (male). **12**Epiphragma (Epiphragma) acuminatum sp. nov. **13**Epiphragma (Epiphragma) henanensis sp. nov. **14**Epiphragma (Epiphragma) insigne (van der Wulp, 1878) **15**Epiphragma (Epiphragma) longitubum sp. nov. Scale bars: 1 mm.

**Female.** Unknown.

#### Material examined.

male (CAU), China: Fujian, Meihuashan, 2006.VIII.31, Hui Dong.

#### Distribution.

China (Fujian), Indonesia (Sumatra), Malaysia (Peninsular, Borneo: Sarawak, Sabah).

#### Remarks.

This species is reported from China for the first time.

### Epiphragma (Epiphragma) longitubum sp. nov

Taxon classificationAnimaliaDipteraLimoniidae

E2F30FA2-6A24-5AFD-98D1-8B4D37F594FF

http://zoobank.org/533DE781-D30B-4811-BBD5-B8A50C08463E

Figs 5 , 6 , 11 , 15 , 34–39

#### Diagnosis.

Generally brown with pale gray pruinosity. Vertex with one median black line. Femora yellow with two dark brown rings, first at basal 2/3, second at tip, broader. Wing brownish hyaline with conspicuous brown pattern, chiefly spotted, with narrow dark brown margin, brownish hyaline areas large. Origin of Rs obtuse and curved with short spur; *m-cu* at basal 1/3 of cell *dm.* Interbase with long and slender rod beyond base, little longer than gonocoxite, whole slender rod straight, tip spine-like. Aedeagus very stubby, 4/5 as long as gonocoxite.

**Figures 16–21.**
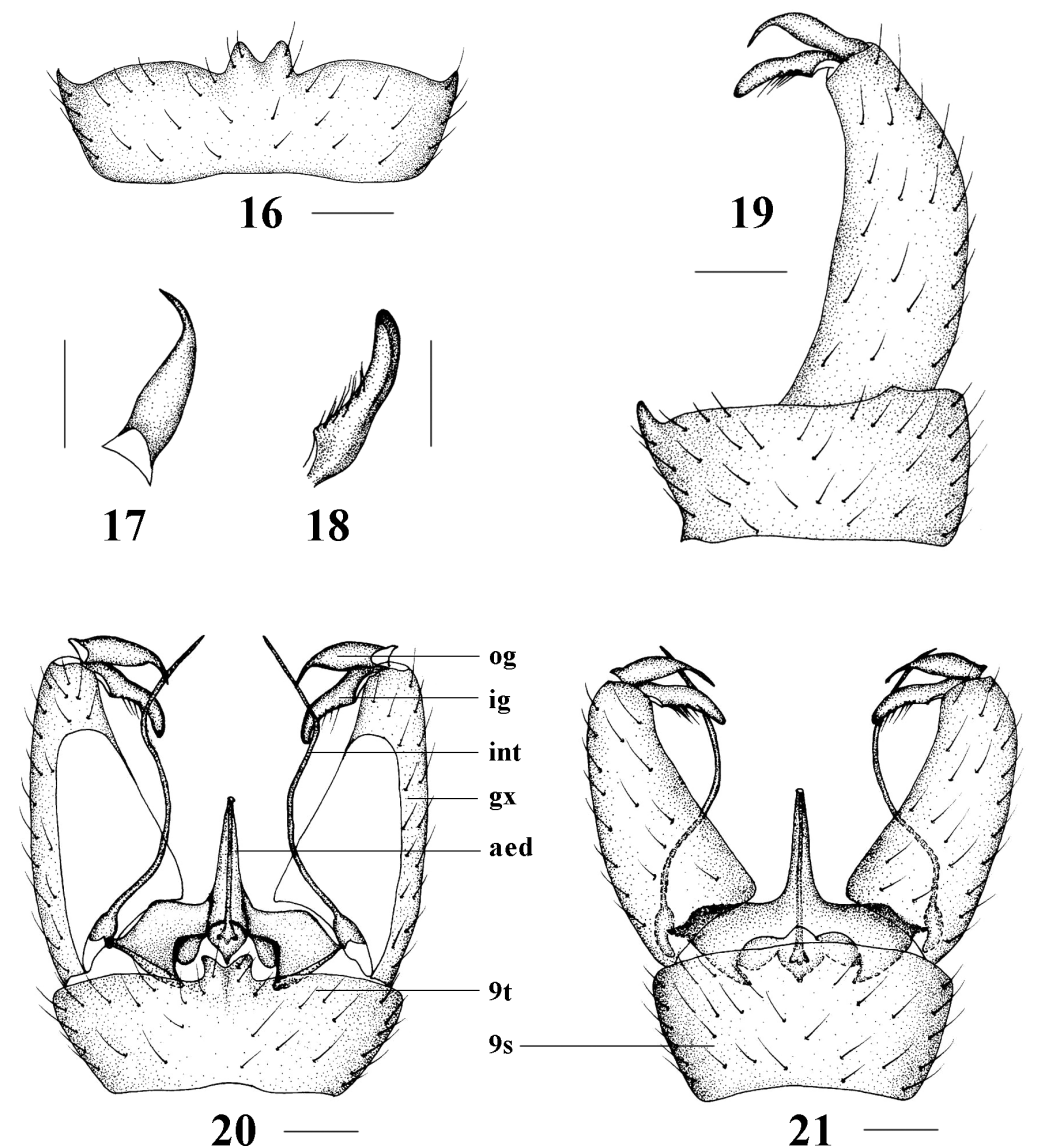
Epiphragma (Epiphragma) acuminatum sp. nov. **16** Ninth tergite, dorsal view **17** outer gonostylus, lateral view **18** inner gonostylus, lateral view **19** hypopygium, lateral view **20** hypopygium, dorsal view **21** hypopygium, ventral view. Scale bars: 0.1 mm.

#### Description.

**Male.** (n = 1): Body length 12 mm, wing length 13 mm, antenna length 3 mm. Head (Figs 5, 11). Brownish yellow with pale gray pruinosity except orbit pale yellow. One median tubercle between eyes. Setae on head black. Scape and pedicel brown, flagellum 13-segmented with two basal segments fused, fusion-segment yellow, succeeding segments brown, flagellomeres cylindrical, apical segments elongate, with longer verticils. Proboscis brownish yellow with black setae; palpus blackish brown with black setae.

Thorax (Figs 5, 11). Generally brown with pale gray pruinosity. Pronotum brownish yellow with one dark blackish brown stripe. Prescutum brown with two brownish yellow stripes, one yellow line at middle. Scutum yellow. Scutellum and mediotergite brownish yellow. Pleura brown, variegated by light yellow areas. Setae on thorax brown. Coxae and trochanters yellow; femora yellow with two dark brown rings, first at basal 2/3, second at tip, broader; tibiae brown with one spur; tarsi brown. Setae on legs brown except coxae and trochanters with brownish yellow setae. Wing (Figs 5, 15). Brownish hyaline with conspicuous brown pattern, chiefly spotted, with narrow dark brown margin, brownish hyaline areas large. Base of wing brown, connected with two large ocellate circles with flattened tips; one circle along cord connected with second circle. Stigma solidly dark brown. Origin of Rs obtuse and curved with short spur; R_2+3+4_ longer than R_2+3_; *m-cu* at basal 1/3 of cell *dm.* Halter 2.1 mm long, brown except base of stem and apex of knob pale yellow. Setae on wings brownish yellow.

Abdomen (Fig. 5). Tergites 1–3 brownish yellow; tergites 4–9 brownish black, with one brownish yellow middle line. Sternites 1–5 brownish yellow; sternites 6–9 brownish black. Setae on abdomen brownish yellow. Hypopygium (Figs 34–39). Posterior margin of tergite 9 (Figs 34, 37, 38) with flat and subtrigonal lobe on each side of median V-shaped notch. Tip of outer gonostylus (Figs 35, 37–39) curved into spine. Inner gonostylus (Figs 36–39) relatively longer, with tip obtuse and curved up. Interbase (Figs 38, 39) with long and slender rod beyond base, little longer than gonocoxite, whole slender rod straight, tip spine-like. Aedeagus (Figs 38, 39) very stubby, 4/5 as long as gonocoxite.

**Female.** (n = 5): Body length 11.0–14.0 mm, wing length 11.5–13.5 mm, antenna length 2.7–3.2 mm. Similar to male. Cerci and hypovalves reddish brown (Fig. 6).

**Figures 22–27.**
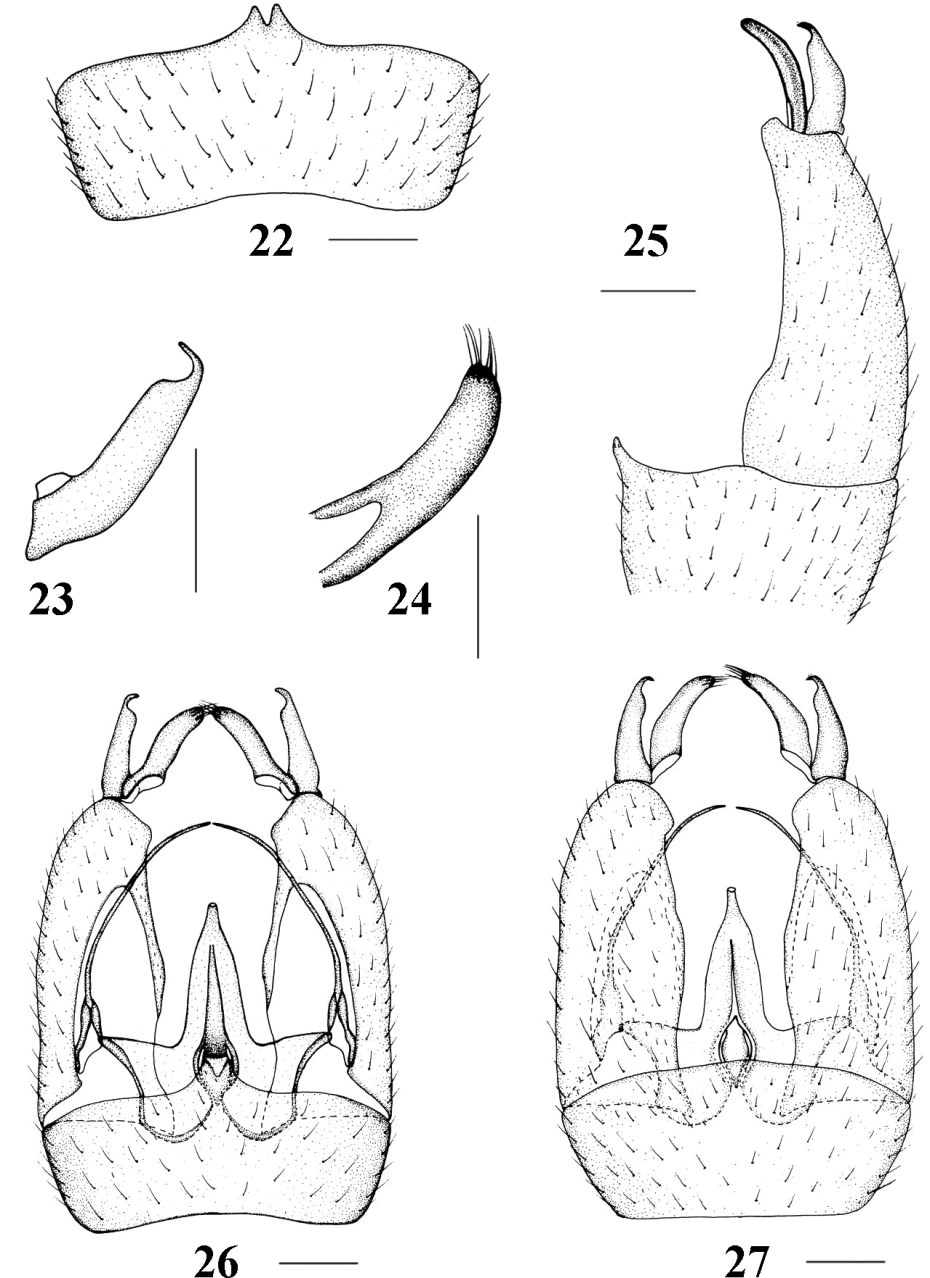
Epiphragma (Epiphragma) henanensis sp. nov. **22** Ninth tergite, dorsal view **23** outer gonostylus, lateral view **24** inner gonostylus, lateral view **25** hypopygium, lateral view **26** hypopygium, dorsal view **27** hypopygium, ventral view. Scale bars: 0.1 mm.

#### Type Material.

**Holotype** male (CAU), China: Guizhou, Tongren, Fanjingshan, 2002.VI.1, Ding Yang. **Paratypes**: 2 females (CAU), China: Guizhou, Tongren, Fanjingshan, 2002.VI.1, Ding Yang; 1 female (CAU), China: Guizhou, Tongren, Fanjingshan, 2002.V.29, Ding Yang; 2 females (CAU), China: Guizhou, Tongren, Fanjingshan, 2002.V.31, Ding Yang.

#### Distribution.

China (Guizhou).

#### Etymology.

The specific name refers to the long aedeagus.

#### Remarks.

This new species is somewhat similar to E. (E.) elongatum Mao & Yang, 2009 from China (Yunnan) in having the similar femora and wing pattern, but can be separated from E. (E.) elongatum by the interbase rod being straight and the aedeagus stubby and 4/5 as long as the gonocoxite. In *E. elongatum*, the interbase rod is finger-shaped, and the aedeagus is slender and half as long as the gonocoxite (Mao and Yang 2009).

**Figures 28–33.**
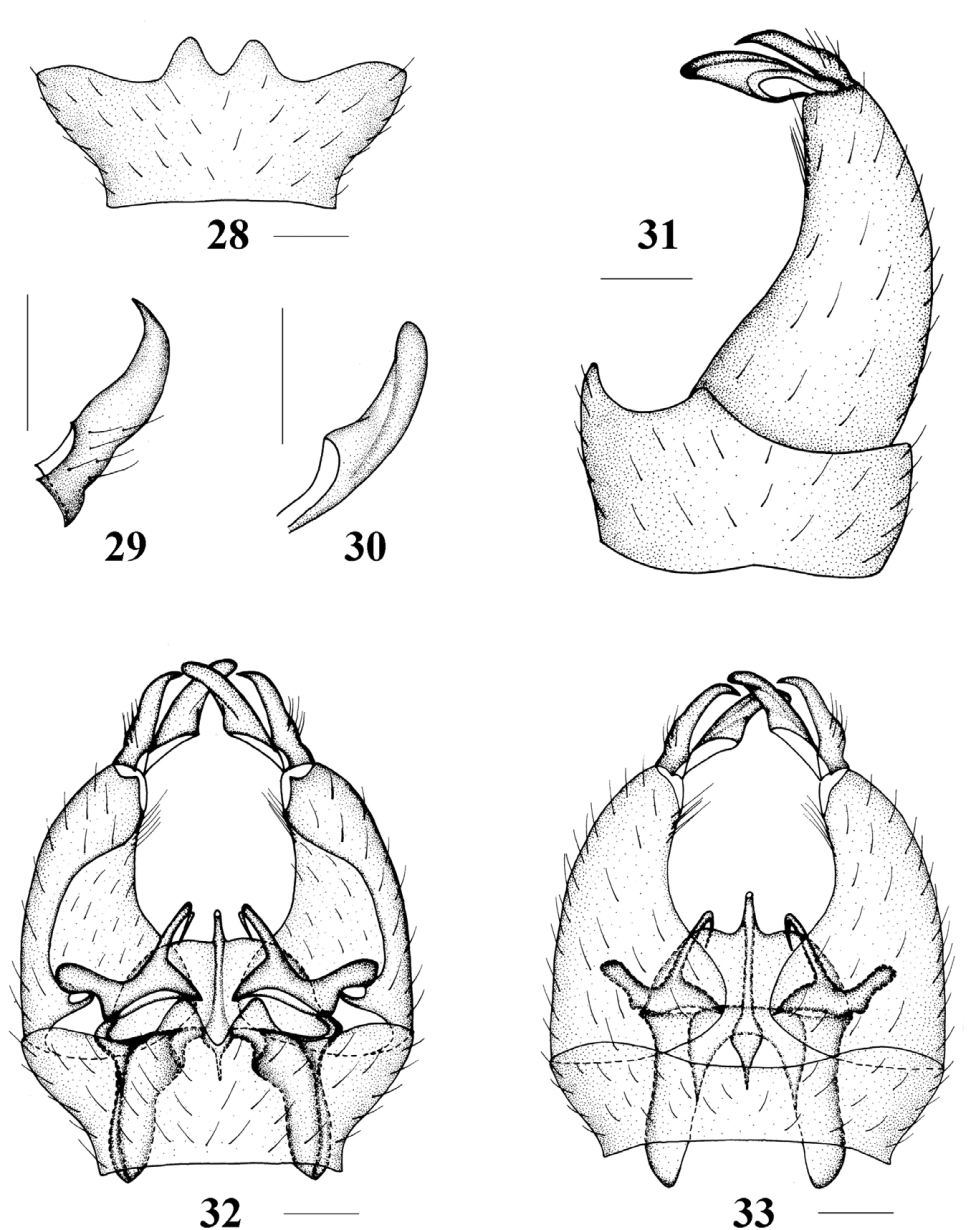
Epiphragma (Epiphragma) insigne (van der Wulp, 1878). **28** Ninth tergite, dorsal view **29** outer gonostylus, lateral view **30** inner gonostylus, lateral view **31** hypopygium, lateral view **32** hypopygium, dorsal view **33** hypopygium, ventral view. Scale bars: 0.1 mm.

**Figures 34–39.**
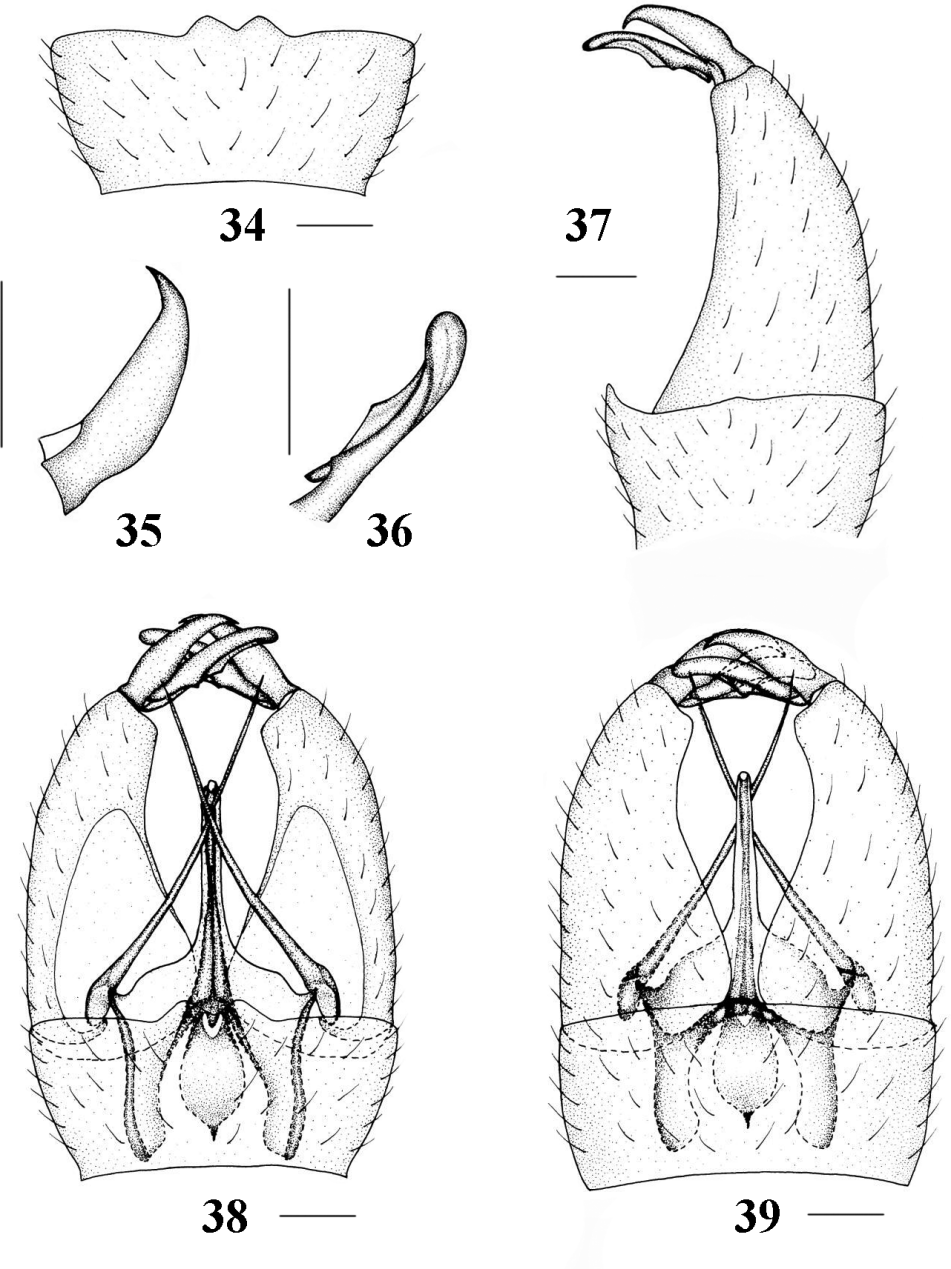
Epiphragma (Epiphragma) longitubum sp. nov. **34** Ninth tergite, dorsal view **35** outer gonostylus, lateral view **36** inner gonostylus, lateral view **37** hypopygium, lateral view **38** hypopygium, dorsal view **39** hypopygium, ventral view. Scale bars: 0.1 mm.

## Supplementary Material

XML Treatment for Epiphragma (Epiphragma) acuminatum

XML Treatment for Epiphragma (Epiphragma) henanensis

XML Treatment for Epiphragma (Epiphragma) insigne

XML Treatment for Epiphragma (Epiphragma) longitubum
